# Morphologic and Genetic Analysis of *Synhimantus* (*Synhimantus*) *laticeps* from a Long-Eared Owl (*Asio otus*)

**DOI:** 10.3390/pathogens12050717

**Published:** 2023-05-15

**Authors:** Carolina Lopes, Sérgio Santos-Silva, Carolina Nunes, Susana Mendes, Catarina Costa, Erica Brazio, Teresa Coutinho, Filipa Teixeira Rodrigues, João R. Mesquita, Ana Cláudia Coelho, Luís Cardoso, Ana Patrícia Lopes

**Affiliations:** 1 Wildlife Rehabilitation Centre of Santo André (CRASSA), Quercus, 7500-022 Vila Nova de Santo André, Portugal; carolina3lopes.vet@gmail.com (C.L.); cnunes94@gmail.com (C.N.); 2 ICBAS—School of Medicine and Biomedical Sciences, Porto University, 4099-002 Porto, Portugal; up202110051@edu.icbas.up.pt (S.S.-S.); jrmesquita@icbas.up.pt (J.R.M.); 3 Exoticvets, 2670-345 Loures, Portugal; susana.mendes@exoticvets.pt (S.M.); catarina.costa@exoticvets.pt (C.C.); 4 Wildlife Rehabilitation Centre of Lisbon (LxCRAS), Parque Florestal de Monsanto, 1050-068 Lisboa, Portugal; erica.brazio@cm-lisboa.pt; 5 Department of Veterinary Sciences, University of Trás-os-Montes e Alto Douro (UTAD), 5000-801 Vila Real, Portugal; tcoutinho@utad.pt (T.C.); filipar@utad.pt (F.T.R.); accoelho@utad.pt (A.C.C.); aplopes@utad.pt (A.P.L.); 6 CECAV—Animal and Veterinary Centre, Associate Laboratory for Animal and Veterinary Sciences (AL4AnimalS), University of Trás-os-Montes e Alto Douro (UTAD), 5000-801 Vila Real, Portugal; 7 Epidemiology Research Unit (EPIUnit), Instituto de Saúde Pública da Universidade do Porto, 4050-600 Porto, Portugal; 8 Laboratório para a Investigação Integrativa e Translacional em Saúde Populacional (ITR), 4050-346 Porto, Portugal

**Keywords:** *Asio otus*, *Synhimantus laticeps*, genetic analysis, morphology, Portugal

## Abstract

The long-eared owl (*Asio otus*) is a medium-sized owl species that is well-distributed in almost all of the territories in Portugal. Nematodes were found in the oral cavity of a long-eared owl (*A. otus*) admitted to CRASSA (Wildlife Rehabilitation Centre of Santo André). During a physical exam and stabilization of the bird, five nematodes were collected. The worms were examined and measured under light microscopy, and photos were taken. After a morphological analysis was conducted, all the nematodes (five females) were identified as *Synhimantus* (*Synhimantus*) *laticeps*. Two specimens were subjected to molecular analysis, which confirmed the result. This study provides a combined morphological and genetic approach to *S. laticeps*. To the authors’ best knowledge, this is the first report including genetic sequencing of *S. laticeps* in a long-eared owl (*A. otus*) from Portugal.

## 1. Introduction

*Synhimantus (Synhimantus) laticeps* (Rudolphi, 1819) Railliet, Henry, and Sisof, 1912, is an acuariid nematode, i.e., it belongs to the family Acuariidae, which is part of the order Spirurida and the superfamily Acuarioidea. The family comprises 25 genera, including *Acuaria*, *Dispharynx*, and *Synhimantus*. The parasite locates in the gastrointestinal tract, namely in the gizzard [1,2], stomach [3], and small intestine [1]. Clinically, a heavy infection with *S. laticeps* may cause ulcers in the proventriculus [4,5]. The species *S. latipes* has been identified in several birds around the world [3], including the black-winged kite (*Elanus caeruleus*), the Eurasian sparrow hawk (*Accipiter nisus*), the barn owl (*Tyto alba*), the long-eared owl (*Asio otus*), the tawny owl (*Strix aluco*), the common kestrel (*Falco tinnunculus*), the common buzzard (*Buteo buteo*), and the Eurasian eagle owl (*Bubo bubo*) [1,5,6,7,8,9]. Despite its widespread distribution, information about this nematode is still scarce. The aim of the present study was to provide a combined morphological and genetic approaches to the *S. laticeps* present in a long-eared owl (*A. otus*) that was admitted to a wildlife rehabilitation center in Portugal. In this report, we give a detailed morphological and morphometric description of *S. laticeps*. We describe the structures that are important for the identification of this parasite and combine that information with the results from a genetic analysis, with both contributing to expanding the scientific knowledge of *S. laticeps*.

## 2. Materials and Methods

### 2.1. Animal and Samples

A long-eared owl (*A. otus*) was admitted to the Wildlife Rehabilitation Centre of Santo André (CRASSA), in southwestern Portugal, with signs of a collision with a structure or vehicle. The animal was very stressed, hypothermic, and severely dehydrated. By palpation, it was possible to identify a fracture involving both metacarpus bones on the right wing. An X-ray was performed, and fractures of both metacarpus bones were confirmed. Both fractures were stabilized with a bandage. The right eye was in mydriasis with no pupillary light reflex, and it was possible to see hyphema. A fluorescein test was performed, and no ulcers were detected. The long-eared owl initially underwent subcutaneous fluid infusion for hydration and nutrition (0.9% sodium chloride, lactated Ringer’s solution, and Duphalyte^®^) and ivermectin (0.4 mg/kg, repeated in 14 days), orally, with meloxicam (0.5 mg/kg, q 24 h), amoxicillin and clavulanic acid (125 mg/kg, q 12 h), tramadol (7.5 mg/kg, q 24 h), and sucralfate (25 mg/kg, q 24 h), and topically, in the right eye, with tobramycin (q 8 h) and dexamethasone (q 8 h). After the first stabilization, diagnostic, and treatment in CRASSA, the animal was transferred to the Wildlife Rehabilitation Centre of Lisbon (LxCRAS) to be submitted to an orthopedic surgery in order to align and stabilize the fracture.

In addition to the traumatic injuries, there were also multiple visible worms and blood in the oral cavity, coming from the esophagus. The helminths were collected, washed in saline solution, fixed, and preserved in 70% alcohol. Then, they were brought to the Laboratory of Parasitology of UTAD (University of Trás-os-Montes e Alto Douro), where morphological and morphometric analyses were made.

### 2.2. Morphological and Morphometric Analysis

For morphological and morphometric analysis, five specimens were mounted in lactophenol, placed on glass slides, and covered with coverslips. They were assessed for their structures using a Leica^®^ (Wetzlar, Germany) DM2000 Led microscope coupled to a digital camera Leica^®^ ICC50 W, with the support of LAS V4.13 software, and photos of important structures were taken. The helminths were identified at the genus level using determination keys [10,11] and other studies for further details [2,8,12]. The acuariid nematodes of terrestrial birds, subfamily Acuariinae, are characterized by cephalic ornamentation, consisting of cordons extending longitudinally and expanding on the cervical region. The cordons of the specimens were long, not enlarged posteriorly, recurrent, and anastomosing [11].

### 2.3. Genetic Analysis

Two specimens were subjected to molecular analysis. Nucleic acid extraction with a customized version of the QIAamp^®^ DNA Mini Kit (Qiagen Inc., Valencia, CA, USA) on an automated platform (QIAcube, Qiagen GmbH, Hilden, Germany) was performed. Parasite DNA detection was performed using the forward primer (designated Nem_18S_F) CGCGAATRGCTCATTACAACAGC (23 bases) and the reverse primer (Nem_18S_R) GGGCGGTATCTGATCGCC (18 bases), targeting a 900 bp region of the 18S small subunit ribosomal RNA gene (18S rRNA) [13]. Nucleic acid amplifications were carried out using Xpert Fast Hotstart Mastermix (2x) with dye (GRiSP, Porto, Portugal), according to the company’s protocol instructions. Briefly, amplification was carried out on 25 μL reaction mix containing 1 μL of each mentioned primer at 10 pmol/μL, 12.5 μL of Xpert Fast Hotstart Mastermix (2x) with dye, 5.5 μL PCR-grade water, and 5 μL of extracted DNA. The PCR thermocycling parameters were established based on the original procedures and modified to suit the commercial PCR kit. Briefly, the initial step involved a cycle of 3 min at 95 °C, followed by 40 cycles consisting of 15 s at 95 °C for denaturation, 15 s at 53 °C for annealing, and 2 s at 72 °C for extension, with a final 3 min extension step at 72 °C. Amplified products of the expected size were purified using GRS PCR and Gel Band Purification Kit (GRiSP) and were bidirectionally sequenced. Consensus sequences were compared with the sequences available in the NCBI (GenBank) nucleotide database “http://blast.ncbi.nlm.nih.gov/Blast” (accessed on 7 October 2022). The MEGA 11 [14] software was utilized for sequence analysis, specifically, the maximum likelihood (ML) method for determining the optimal substitution model. The phylogenetic tree was generated using the Kimura 2-parameter model with the assumption that rate variation among sites was gamma distributed. A bootstrap consensus tree was created from 1000 pseudoreplicates. The final phylogenetic tree was styled using the Interactive Tree of Life (iTOL) platform [15].

## 3. Results

### 3.1. Morphological and Morphometric Analysis

**Figure 1:**
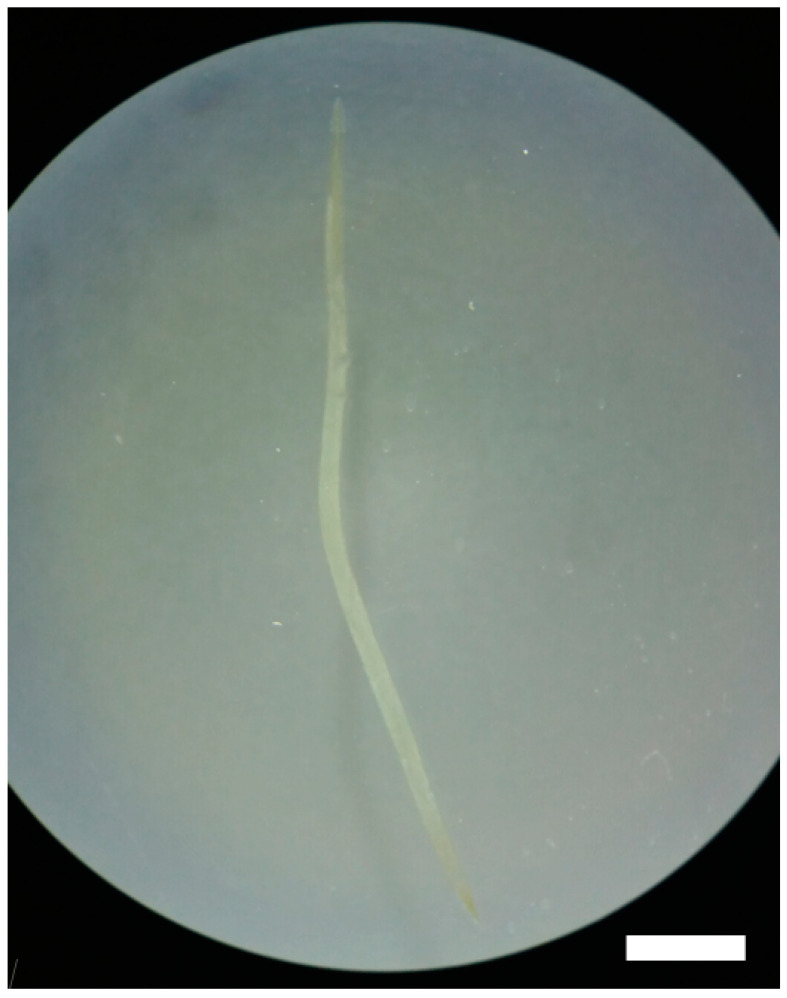
Female *Synhimantus laticeps*. Scale bar: 3.0 mm.

**Figure 2:**
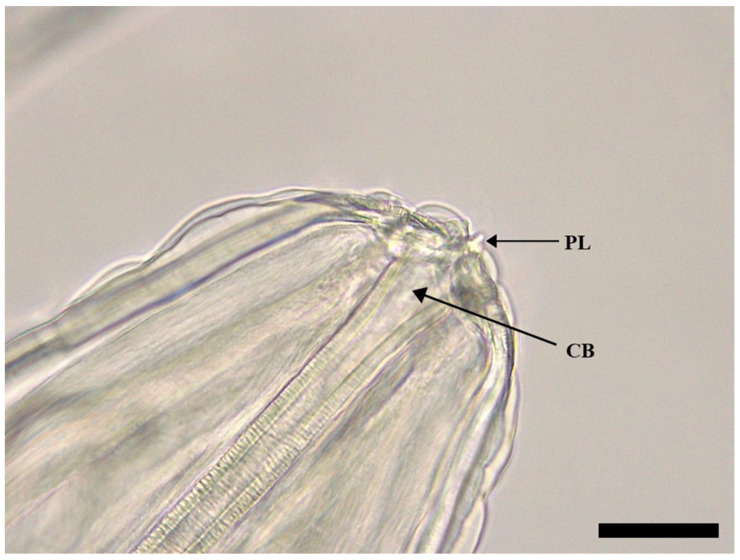
Female of *Synhimantus laticeps*: apical view of the pseudolabia (PL) and buccal capsule (BC). Scale bar: 50 μm.

**Figure 3:**
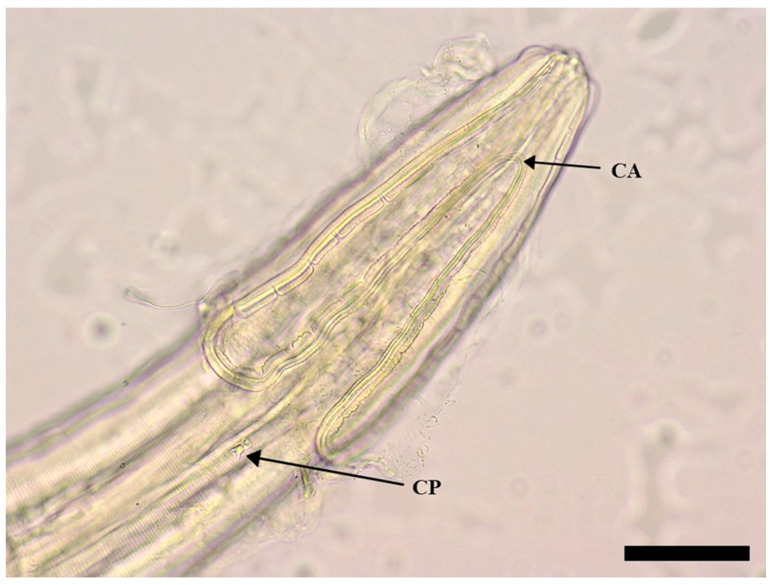
Female of *Synhimantus laticeps*: anterior region and lateral view, with cordons recurrent and anastomosed (CA) and cervical papillae (CP). Scale bar: 200 μm.

**Figure 4:**
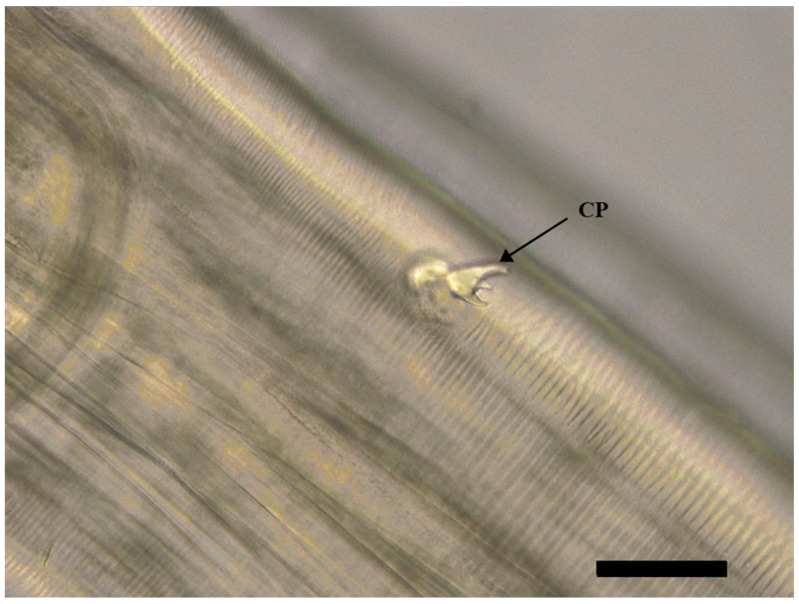
Female of *Synhimantus laticeps*: cervical papillae tricuspid (deirids) (CP) and lateral view, showing the typical tridentate shape. Note cuticle covered with fine transverse striations. Scale bar: 50 μm.

**Figure 5:**
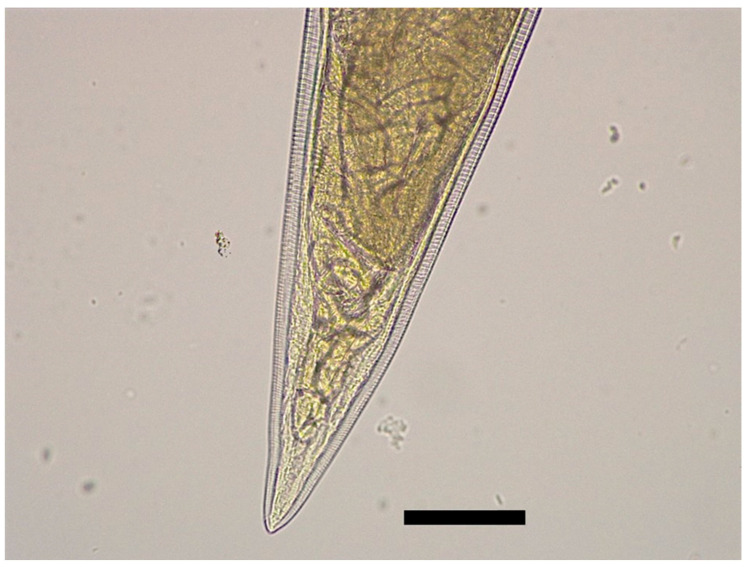
Female of *Synhimantus laticeps*: posterior end of female. Scale bar: 200 μm.

**Figure 6:**
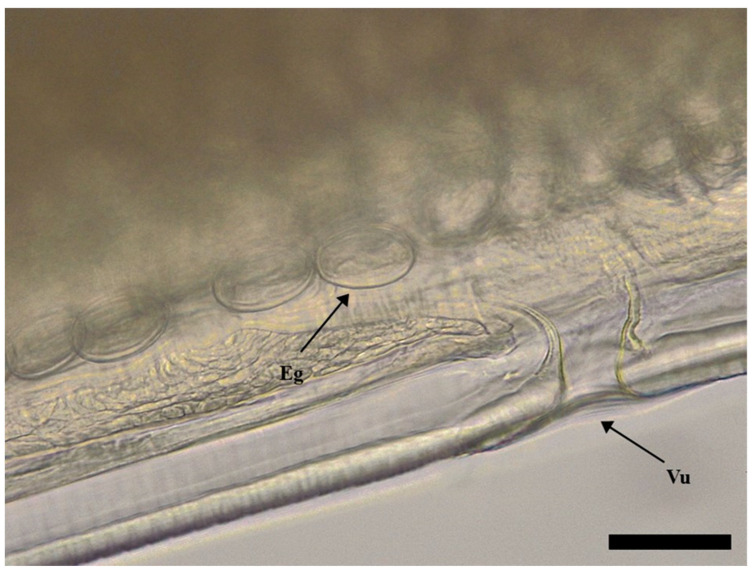
Female of *Synhimantus laticeps*: detail view of vulva (Vu) and larvated eggs (Eg). Scale bar: 50 μm.

The body of *S. laticeps* had a yellow and whitish color (Figure 1), and the cuticle was covered with fine transverse striations. On the anterior part, two lateral pseudolabia were visible, each of them with a small process tapered conically. The buccal capsule was narrow and long, and it expanded anteriorly (Figure 2). Arising from the lateral margins of the pseudolabia, the cutaneous cordons extended caudally, reaching a certain distance, and then they returned and recurred to more than one-half their length, anastomosing in pairs on each lateral surface (Figure 3). The cervical papilla (deirids) was located posteriorly to the cordons on each side and had a typical tridentate shape (Figure 3 and Figure 4). The tail was conical, and a small inconspicuous vulva was located near the middle of the body (Figure 5 and Figure 6).

**Figure 7:**
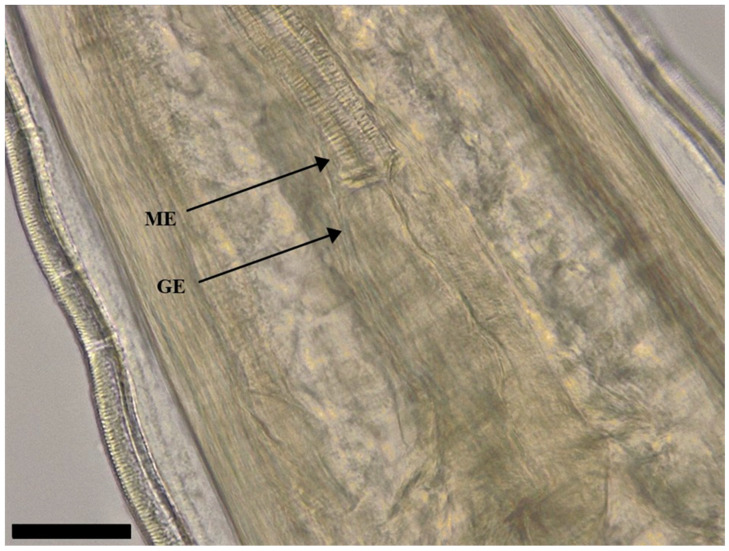
Female of *Synhimantus laticeps*: anterior end of muscular esophagus (ME) and opening of the glandular esophagus (GE). Scale bar: 50 μm.

Females (the average and minimum–maximum (min–max) measurements of five specimens; Table 1): the average body length was 19.8 (min–max: 19.7–19.9) mm and the width was 547.68 (min–max: 535.84–559.52) µm. The length of the muscular esophagus was 440.78 µm and the glandular esophagus was 1.01 mm long (Figure 7). Cordons were 661.74 (min–max: 480.82–734.24) µm and 148.01 (min–max: 127.31–174.58) in width (widest part). Deirids were located at a 717.77 (min–max: 540.1–799.28) µm distance from the anterior extremity (dfae), with a diameter of 27.53 (min–max: 23.3–33.5) µm and 23.63 (min–max: 17.62–33.54) µm long. Larvated oval-shaped eggs, thick-shelled, contained in the uterus were 39.54 (min–max: 38.71–40.36) in length and 24.32 (min–max: 24.23–24.40) in width.

Comparative measures from the present study with other authors are presented in Table 2.

### 3.2. Genetic Analysis

**Figure 8:**
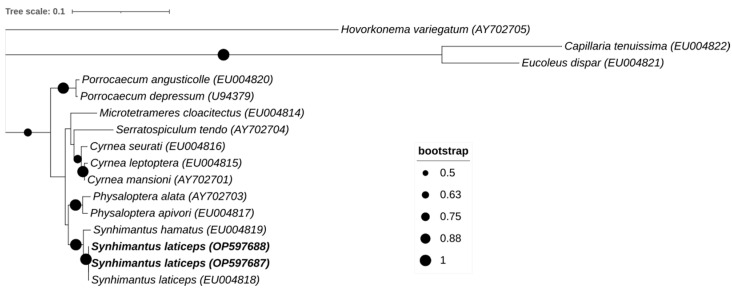
Phylogenetic analysis of retrieved *Synhimantus laticeps* sequences (alignment length: 715 bp). The tree was inferred using the MEGA 11 maximum likelihood method (Kimura 2-parameter) and the Interactive Tree of Life (iTOL) based on 16 nucleotidic sequences of the 18S rRNA, including sequences found in this study (*S. laticeps*, plus its accession numbers, are in bold) and sequences from different species obtained from GenBank (no bold or shading and identified by their accession number).

Regarding the genetic analysis, the BLAST sequence analysis of the consensus 18S small subunit ribosomal RNA sequences revealed both samples to be 100% identical with *S. laticeps* (accession number KP861914) isolated from a sparrowhawk (*Accipiter nisus*) from the Netherlands and a *S. laticeps* (accession number EU004818) isolated from a free-ranging bird from Germany. The 18S rRNA sequences of *S. laticeps* obtained in this study are available at GenBank under accession numbers OP597687 and OP597688. Phylogenetic analysis confirmed that both sequences were *S. laticeps* (Figure 8).

## 4. Discussion

The long-eared owl (*A. otus*) is a medium-sized species of owl of the family Strigidae that breeds in many areas, but mostly in Europe and North America [16]. In Portugal, this nocturnal raptor may be seen almost every year throughout the country [17]. The long-eared owl (*A. otus*) is an adaptable predator that expands its food niche in the presence of diversified prey [18]. Its overall diet is dominated by small mammals. However, in Italy, the monthly variation showed that the owls fed on three main categories of prey: *Apodemus* spp., *Microtus* spp., and birds [18].

Previous studies have shown that nematodes of the genus *Synhimantus* are prevalent helminths in the proventriculus of raptors [1,3,8,19]. Infections of *S. laticeps* of greater intensity (23 ± 36.8) and abundance (7.7 ± 22.3) were found in birds of prey in Spain [1]. Additionally, a study in the south of Spain reported that *S. laticeps* was the second-most prevalent helminth in 50 Eurasian eagle owls (*Bubo bubo*) [9]. Even though there are some reports of *S. laticeps* in raptors, including one from Portugal [8], the life cycle of this parasite is still not fully understood. However, since the adult stages mainly occur in birds of the orders Accipitriformes, Falconiformes, and Strigiformes, it has been hypothesized that small vertebrates could be involved as intermediate hosts [10]. Nevertheless, the possible development of acuariid nematodes from arthropod intermediate hosts such as grasshoppers, beetles, and diplopods has also been suggested [20].

The morphological features obtained in the present study are in line with previous reports. In Portugal, Tomás et al. [8] described the presence of *S. laticeps* in the gizzard of a black-winged kite (*Elanus caeruleus*) in a morphological analysis. One of the most important structures for the identification of *S. laticeps* is the presence of cutaneous cordons anastomosed on the lateral surface [3,8]. All the measurements obtained in the present report are in line with others, regarding the length and width of specimens, cordons, deirids, and larvated eggs, as well as the measurements of the esophagus (muscular and glandular) [2,3,12,21].

Acosta et al. [6] measured the length of 15 males of *S. laticeps* and obtained a medium value of 8.89 ± 1.17 (6.83–10.60) mm. In the present study, two females were measured, with a value of 19.8 mm, which agrees with other studies [3,12] that obtained larger dimensions in females, regarding length and width, when compared to the only male included in the studies. Considering the scarcity of the existing data, the measurements carried out in this study corroborate previous data and add new information for a more rigorous and reliable morphological characterization of *S. laticeps*. It may be suggested that females of *S. laticeps* have bigger dimensions than males.

To our knowledge, this is the first report, including genetic confirmation (Figure 8) of *S. laticeps* in a long-eared owl (*A. otus*) in Portugal. There is little information about this parasite in terms of genotyping [12,22,23]. A study was carried out in free-ranging birds from Germany [22], and the obtained sequence was submitted to GenBank (accession number EU004818), which is 100% identical to the 18S rRNA sequences of *S. laticeps* obtained in the present study, available at GenBank under accession numbers OP597687 and OP597688. The phylogenetic analysis of the 18S rRNA sequences obtained in this study confirmed the classification as *S. laticeps*.

Some authors consider *Dispharynx* to be a subgenus of the genus *Synhimantus*, which can be theoretically differentiated from *S. laticeps* by the anastomosed cordons in the latter [24]. However, morphological differentiation is sometimes difficult, and the use of genomic techniques may be very helpful, including in subadults and larval stages where the morphological structures are not completely differentiated, and genetic sequencing may be necessary to identify the parasite [21]. In the study by Honisch and Krone [22], phylogenetic relationships and the traditional morphological systematics of Spiruromorpha from birds of prey were compared, and a good agreement was observed between the morphological systematics and the constructed 18S phylogenetic tree.

## 5. Conclusions

The present study consists of a morphological and morphometric analysis of *S. laticeps* and includes detailed photos of the structures deemed important, which is a circumstance that contributes to the correct identification of *S. laticeps* under light microscopy. To the authors’ best knowledge, this is the first report including genetic sequencing of *S. laticeps* in a long-eared owl (*A. otus*) from Portugal. This study provides additional and updated information on *S. laticeps* and highlights the need for further studies to be conducted to better understand its global distribution, hosts, and morphological and genetic features.

## Figures and Tables

**Table 1:** Measurements (in µm unless otherwise indicated) of five female specimens of *Synhimantus laticeps* from a long-eared owl (*Asio otus*).

	Specimen 1	Specimen 2	Specimen 3	Specimen 4	Specimen 5
Length (mm)	na	na	na	19.7	19.9
Width	na	na	na	559.52	535.84
Cordons length	731.14	480.82	724.60	637.88	734.24
Cordons width	127.31	159.89	174.58	133.148	145.11
Muscular esophagus	na	na	na	na	440.78
Glandular esophagus (mm)	na	na	na	na	1.01
Deirids (dfae)	802.3	540.1	729.4	763.43	799.28
Deirids length	23.31	33.54	na	17.62	20.03
Deirids width	23.3	33.5	na	na	25.8
Larvated eggs in length *	na	na	na	40.36	38.71
Larvated eggs in width *	na	na	na	24.23	24.40

dfae: distance from anterior extremity; na: not available; * average measurements of three eggs for each female.

**Table 2:** Comparative measurements (in µm unless indicated otherwise) of female specimens of *S. laticeps*.

References	Etchegoin et al. (2000)	Umur et al. (2010)	Ebmer et al. (2017)	Asti et al. (2017)	Present Study
Geographic distribution	Argentina	Turkey	Austria	Turkey	Portugal
Host	Barn owl	Sparrowhawk	Barn owl	Common kestrel	Long-eared owl
	(*Tyto alba*)	(*Accipiter nisus)*	(*Tyto alba*)	(*Falco tinnunculus*)	(*Asio otus)*
Number of specimens (n)	2	1	5	12	5
Length (mm)	4.85	1.50	7.74	6.28–10.86	19.8 ^#^
Width	215	480	250	na	547.68 ^#^
Cordons length	146	635.64	296	555.96–1412.04	661.74
Cordons width	na	314.17	199	na	148.01
Buccal capsule	108	269.38	na	na	na
Muscular esophagus	405	908.18	490	497.84–1084.27	440.78 *
Glandular esophagus (mm)	2.11	3.35	2.31	2.01–3.37	1.01 *
Deirids (dfae)	233	712.71	na	na	717.77
Deirids length	14	21.31	na	na	23.63 ^£^
Deirids width	na	na	15.3	na	27.53 ^$^
Larvated eggs in length	35.6	37.66	37.3	36.90–44.28	39.54 ^#^
Larvated eggs in width	20	23.98	23.9	22.14–24.60	24.32 ^#^

dfae: distance from anterior extremity; *: n = 1; ^#^: n = 2; ^$^: n = 3; ^£^: n = 4; na: not available.

## Data Availability

Data supporting the conclusions of this article are included within the article and its additional file. The datasets used and/or analyzed during the present study are available from the corresponding author on reasonable request.
